# The Alpha and Beta Diversities of Dietary Patterns Differed by Age and Sex in Young and Middle-Aged Japanese Participants

**DOI:** 10.3390/nu17132205

**Published:** 2025-07-02

**Authors:** Katsumi Iizuka, Kotone Yanagi, Kanako Deguchi, Chihiro Ushiroda, Risako Yamamoto-Wada, Takuma Ishihara, Hiroyuki Naruse

**Affiliations:** 1 Department of Clinical Nutrition, Fujita Health University, Toyoake 470-1192, Japan; kanasakuran@gmail.com (K.D.); chihiro.ushiroda@fujita-hu.ac.jp (C.U.); risako.wada@fujita-hu.ac.jp (R.Y.-W.); 2 Food and Nutrition Service Department, Fujita Health University Hospital, Toyoake 470-1192, Japan; 3 Health Management Center, Fujita Health University, Toyoake 470-1192, Japan; yanagi-k@fujita-hu.ac.jp (K.Y.); hnaruse@fujita-hu.ac.jp (H.N.); 4 Innovative and Clinical Research Promotion Center, Gifu University Hospital, Gifu 501-1194, Japan; ishihara.takuma.d0@f.gifu-u.ac.jp

**Keywords:** Shannon index, nonmetric multidimensional scaling, NMDS, redundancy analysis, RDA, dietary patter, sex difference, age difference, food intake frequency

## Abstract

**Background/Objectives**: Dietary patterns vary with age and sex. The aim of this study was to clarify the differences in dietary patterns among young and middle-aged Japanese individuals by age group and sex via statistical methods such as alpha diversity and beta diversity analyses. **Methods**: Using data from a dietary survey of 10 food items during health checkups of 2743 Fujita Health University employees, we examined the effects of age and sex on alpha diversity (Shannon index) and beta diversity (nonmetric multidimensional scaling (NMDS) and RDA). Unlike principal component analysis which assumes linear relationships, redundancy analysis (RDA) incorporates explanatory variables to directly assess how external factors shape multivariate patterns. **Results**: The Shannon index increased with age and was greater in males across age groups. Type III ANOVA revealed significant main effects of age (*p* < 0.001) and sex (*p* < 0.001), and the effect of the interaction between age and sex approached significance (*p* = 0.08). Visualization of the NMDS data revealed that women aged 20–29 years and women aged 30 years and older and men aged 20–39 years and men aged 50–59 years have different dietary patterns. The RDA model accounted for 2.01% of the variance (adjusted R^2^ = 1.94%), with age and sex contributing 56.7% and 43.3%, respectively. RDA1 and RDA2 were correlated with age (r = 0.26, −0.14) and sex (r = 0.15, 0.21). The RDA1 values increased with age and were greater in females, whereas the RDA2 values decreased with age and were greater in females. RDA1 (1.41% of the total variance in food group intake, 70.1% of the constrained variance) was positively associated with fruits, milk, and seaweed and negatively associated with meat and eggs. In RDA2 (0.60% of total variance, 29.9% contribution), fruits, potatoes, and vegetables had positive effects, whereas fish had negative effects. **Conclusions**: Dietary patterns vary by age and sex, with meat, fish, eggs, and fruit as key determinants. Nutritional guidance must account for variations in dietary patterns influenced by age and sex.

## 1. Introduction

It is empirically evident that dietary preferences vary with age and sex [1,2]. For example, taste preferences vary with age and sex [1,2]. In terms of food intake, men tend to eat more meat, whereas women prefer fish [3,4]. Sex-based differences have also been observed in the consumption of fruits [3,4]. Furthermore, the foods consumed also differ by age [5,6]. Analysis of the quantitative index for dietary diversity in the Japanese population has shown that dietary diversity increases in men in their 40s and women in their 50s and decreases in both sexes after 60 years of age [5]. Many previous studies have used the dietary diversity score (DDS) or the QUANTIDD [5,6,7]. However, given the issues associated with food mixing and matching in diets, the DDS or QUANTIDD cannot adequately capture dietary patterns. Because the QUANTIDD (also called the Cao1 index) is a measure of diversity [8], it is specialized for estimating the number of species. Thus, although the frequency of the consumption of certain foods can be determined to some extent, it is necessary to consider dietary patterns because foods are consumed in combination with each other. Therefore, we considered it necessary to develop an analytical method that differs from DDS and QUANTIDD.

Diversity is a parameter that is used to examine gut bacteria and biological ecology [9,10]. The types of diversity include alpha diversity (diversity within a single community or sample) and beta diversity (differences among multiple samples) [9,10,11,12,13,14]. The parameters of alpha diversity (diversity within a single community or sample) include the Shannon diversity index (Shannon index), which considers the number and evenness of species, and the Simpson diversity index (Simpson index), which is strongly influenced by dominant species [11,12]. The methods used to examine beta diversity (differences among multiple samples) include principal coordinate analysis (PCoA) [12], which visualizes the similarities and differences among samples in two or three dimensions from a distance matrix (PCoA/MDS) [12]. Nonmetric multidimensional scaling (NMDS) is a technique that uses a dissimilarity or distance matrix to represent objects in multidimensional space, with a focus on preserving the rank order of dissimilarities rather than the exact distance values [13]. In addition, redundancy analysis (RDA) is a type of multivariate analysis that simultaneously explains multiple objective variables (e.g., microbiome patterns) with multiple explanatory variables [14]. RDA is characterized by the ability to evaluate differences in intake patterns after adjusting for multiple explanatory variables [14]. Although this method is used relatively often in studies of the gut microbiota, it has rarely been used to analyze dietary patterns.

The dietary patterns of Japanese people and age-specific differences in the frequency of individual foods have been examined. For example, meat intake among Japanese individuals is high among 15–19-year-olds and declines thereafter. Fish and vegetable intake increases with age, with the highest intake reported for those aged 60–69 years [15]. Principal component analysis (PCA) has been used in many cases to analyze the dietary patterns of Japanese people [16]. They conducted a meta-analysis of 80 articles and reported that healthy Japanese dietary patterns were rich in mushrooms, seaweeds, potatoes, fruits, legumes, and pickles [16]. However, because PCA depends on the dietary patterns of the population, it is not possible to examine the effects of age and sex. However, since the dietary patterns of Japanese individuals differ by age and sex, it is necessary to consider these effects. Therefore, since RDA can set age and sex as explanatory variables and dietary pattern as a response variable [17], we thought it would be possible to analyze the effects of age and sex on dietary patterns.

On the basis of the results of a food intake frequency survey conducted during medical examinations of employees aged 20–59 years (*n* = 2743), we aimed to clarify the differences in dietary patterns among young and middle-aged Japanese individuals by age group and sex via alpha diversity and beta diversity analyses. First, we evaluated the within-group diversity of dietary intake via alpha diversity indices, and we evaluated the interaction between age and sex. Next, we visualized the overall dietary pattern distributions via NMDS. As the Shannon index and NMDS also revealed that the distance between groups was divided by age and sex, we conducted RDA adjusting for age and sex to identify the contribution of individual food to the RDA axes. Our results indicated that the dietary patterns of the groups differed by age and sex and were defined by the frequency of intake of meat, fish, eggs, fruits, and dairy products. Thus, age and sex define dietary pattern differences, and dietary guidance that accounts for differences in dietary patterns by age and sex is necessary.

## 2. Materials and Methods

### 2.1. Study Design and Participants

This retrospective cross-sectional observational study aimed to clarify the differences in eating patterns with respect to sex and age. The target population (*n* = 2743) consisted of 921 men and 1822 women who had undergone a medical checkup by 2024 and had responded to the dietary questionnaire. The physical data and food frequency questionnaires were provided by the healthcare center at our university in a fully anonymized form, so the data were depersonalized (accessed on 26 April 2025). In this study, we analyze data that has already been anonymized, making it impossible to identify individuals. For this reason, we have announced on our website of the Department of Clinical Nutrition, Fujita Medical School (the approval date: from 21 April 2025 to 31 March 2027) that exclusion is not possible. The study was conducted according to the principles of the Declaration of Helsinki and approved by the Research Ethics Committee of Fujita Health University (application number HM24-581, 21 April 2025).

### 2.2. Detailed Physical Examination

Age (years), sex (M:0, F:1), body mass index (BMI, kg/m^2^), and blood parameter (glycated hemoglobin A1c (HbA1c,%), estimated glomerular filtration rate (eGFR, mL/min/cm^2^), total cholesterol (TC, mg/dL), triglyceride (TG, mg/dL), and high-density lipoprotein cholesterol (HDL-C, mg/dL)) data at the health checkup were obtained from the Health Management Department as anonymized data linked to the FFQg. Height and body weight were measured in the presence of a nurse [4]. BMI was automatically calculated from height and weight. Sleep duration was self-monitored, and the subjects simply listed the average sleep duration per week (e.g., six hours). The plasma creatinine and lipid (TC, TG, and HDL-C) concentrations were measured via a Hitachi LABOSPECT008 (Hitachi High-Tech Corporation, Tokyo, Japan) and HbA1c was measured via an A1c HA-8190 (Arkray, Kyoto, Japan). The eGFR was automatically calculated from the plasma creatinine level, age, and sex. Non-HDL-C concentrations were calculated from the TC and HDL-C concentrations. Blood samples were collected from non-fasting participants at approximately 16:00–17:00. As this was a hospital staff medical checkup, it was set at 3:30 p.m. when the medical staff had finished seeing the outpatients. The data are presented as the means (SDs).

### 2.3. Food Intake Frequency Questionnaire on Food Groups

During the health checkup, the FFQg was used to assess food intake frequency for employees at Fujita Health University every year [4]. The FFQg is one of the most widely used food intake frequency questionnaires in Japan [4,18] and includes questions on the frequency of eating 10 different food types (meat, fish, egg, dairy product, soy, green vegetable (vegetable), seaweed, fruit, potato, and oil); the frequency of drinking sugar-sweetened coffee and tea; and the frequency of consuming soft sweets, colas, and other soft drinks and alcohol. For the ten food types, the subjects simply listed how many times per week they had consumed each type of food. Data are presented as the median (interquartile range, IQR).

### 2.4. Statistical Analysis

#### 2.4.1. Alpha Diversity Indices

Dietary alpha diversity was assessed via the Shannon and Simpson indices, which were calculated for each participant. Differences among the eight groups divided by age (20–29, 30–39, 40–49, 50–59 years) and sex (male/female) were analyzed. The Shannon diversity index for each sample was calculated in Excel via the formula −∑(pi⋅ln(pi)). To avoid errors from ln(0), zero values were replaced with 0.001 as a correction. To analyze differences in the Shannon index, we used R software (version 4.5.0 (2025-04-11); The R Foundation for Statistical Computing, Vienna, Austria) and RStudio (version 2025.05.0+496). The following packages were used: dplyr (v1.1.2) for data handling, car (v3.1-2) for Type III ANOVA, and ggplot2 (v3.4.2) for visualization. The Shannon index was set as the dependent variable. Age group and sex were treated as categorical independent variables (factors). A linear model including main effects and the interaction term (age × sex) was constructed. Type III analysis of variance was performed via the ANOVA function in the car package. Boxplots were generated with ggplot2 to visualize the distribution across groups, as appropriate. The data are presented as the means with 95% confidence intervals (CIs). *p* < 0.05 was considered to indicate statistical significance.

#### 2.4.2. Visualization and Multivariate Analysis

To visualize dietary patterns, nonmetric multidimensional scaling (NMDS) was performed via standardized food group intake data via R software (version 4.5.0 (2025-04-11); The R Foundation for Statistical Computing, Vienna, Austria) and RStudio (version 2025.05.0+496). The following R packages were used: vegan (v2.6–4), dplyr (v1.1.2), ggplot2 (v3.4.2), and boot (v1.3–28.1). Dietary intake data for 10 food groups (meat, fish, egg, soy, milk, seaweed, vegetable, fruit, potato, and oil) were used, and groups were defined by combinations of age and sex (e.g., “1-0”). Bray–Curtis dissimilarity matrices were calculated via the vegdist function, and NMDS was performed in two dimensions (metaMDS function, k = 2, trymax = 100). For each age–sex group, the centroid (mean NMDS1 and NMDS2 values) and 95% confidence intervals were estimated via nonparametric bootstrapping with 2000 replicates (boot package). Plots were generated via ggplot2, which shows group centroids and 95% confidence intervals, either with group colors or in black. Data are represented as medians of centroids with 95% CIs.

#### 2.4.3. Redundancy Analysis (RDA)

All multivariate analyses were conducted via R software (version 4.5.0 (2025-04-11); The R Foundation for Statistical Computing, Vienna, Austria) and RStudio (version 2025.05.0+496). The following packages were used: vegan (v2.6-4) for RDA, boot (v1.3–28.1) for bootstrap estimation, dplyr (v1.1.2) for data manipulation, and ggplot2 (v3.4.2) and ggrepel (v0.9.3) for data visualization. The dietary intake data for 10 food groups were standardized via Z score transformation and used as dependent variables. The RDA model was constructed with ‘rda(Y~Age + Sex)’, and sample scores (scaling = 2) were extracted. Group centroids based on age–sex combinations were computed via 2000 bootstrap replicates (boot package), and 95% confidence intervals were defined as the 2.5th and 97.5th percentiles. The RDA biplot was created via ‘ggplot2’, visualizing both sample scores (sites) and food loadings (species) with scaling adjustment (e.g., multiplied by 0.3–2.5 for clarity). The arrows indicate food contributions, and the group centroids are overlaid with confidence intervals. In some plots, the biplot scores of explanatory variables (from ‘display = “bp”’) were also visualized as vectors to illustrate the influence of age and sex. Data are represented as centroids of the median with 95% CI.

To assess between-group differences along the first and second RDA axes (RDA1 and RDA2), Kruskal–Wallis tests were first conducted. When significant differences were found, post hoc comparisons were performed via Dunn’s test with Bonferroni correction (‘dunnTest()’ function from the ‘FSA’ package). In addition to *p* values, effect sizes were calculated via Cliff’s delta for all pairwise group comparisons (‘cliff.delta()’ function from the ‘effsize’ package). This nonparametric metric quantifies the magnitude of the difference and provides 95% confidence intervals. The results were d in matrix heatmaps to highlight both the direction and magnitude of the effects. *p* < 0.05 was considered to indicate statistical significance. According to Romano et al. (2006), the magnitude of Cliff’s delta (|δ|) can be interpreted as follows: values between 0.00 and 0.147 indicate a negligible effect, values from 0.147 to 0.33 represent a small effect, values from 0.33 to 0.474 indicate a medium effect, and values equal to or greater than 0.474 are considered large effects [19].

## 3. Results

### 3.1. Background of the Participants in This Study

To determine differences in dietary patterns by age and sex, we first aimed to examine the basic characteristics of the study participants (*n* = 2743; male (*n* = 921) and female (*n* = 1822)) (Table 1). The overall age [mean (SD)] of the participants was 36.05 (11.39) years, with values of 38.24 (10.73) years for males and 34.94 (11.56) years for females; the mean age of the male participants was significantly greater than that of the female participants. The overall BMI [mean (SD)] was 22.05 (3.45), with values of 23.30 (3.45) for males and 21.42 (3.27) for females; the mean BMI of the male participants was significantly greater than that of the female participants. The blood parameters of the cohort were as follows: HbA1c [mean (SD)]: 5.5 (0.4)%; eGFR [mean (SD)]: 89.8 (17.9) mL/min/1.73 m^2^; total cholesterol [mean (SD)]: 190.3 (32.4) mg/dL; triglyceride [mean (SD)]: 105.3 (86.3) mg/dL; HDL-C [mean (SD)]: 62.5 (14.5) mg/dL; and UA [mean (SD)]: 4.8 (1.3) mg/dL (Table 1). These values of body size and blood parameters were close to the normal ranges.

### 3.2. Individual Food Frequencies of the Participants in This Study

Next, with respect to the frequency of individual food intake, there were statistically significant differences between males and females in the frequencies of consumption of meat, fish, soybeans, dairy products, vegetable, fruit, and potato (meat: <0.001, fish: <0.001, soy: 0.0036, dairy products: <0.001, vegetable: 0.0059, fruit: <0.001 potato: <0.001), but the effect size (Cliff’s delta) was negligible (meat: 0.099; fish: 0.11; egg: 0.005; soy: −0.068; dairy product: −0.11; seaweed: −0.039; vegetable: −0.064; fruit: −0.19; potato: −0.10; oil: −0.0007). Only a few fruits were consumed 2 (0~4) times/week by males and 3 (1~5) times/week by females, and the effect size (Cliff’s delta) was −0.19, which was small (Table 2). The frequencies of egg, seaweed, and oil consumption were not significantly different between males and females (*p* = 0.86, 0.086, and 0.98, respectively) and Cliff’s delta was negligible (egg: 0.05; seaweed: −0.039; oil: −0.0007) (Table 2). Thus, although the difference in food intake frequency between men and women was significant in terms of *p* values, the effect size (Cliff’s delta) was almost negligible, suggesting that differences in the frequency of individual food intake between males and females were very small or negligible.

### 3.3. Alpha Diversity by Age and Sex

Differences in the frequency of individual food consumption were observed between male and female participants. To further explore these differences, the Shannon index, a measure of alpha diversity, was used to compare the frequency and uniformity of consumption patterns for the ten foods analyzed in this study across sex and age groups.

**Figure 1:**
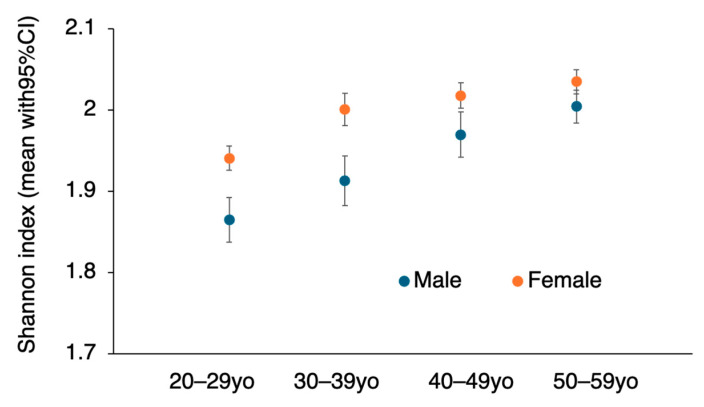
Effects of age and sex on the Shannon index. The Shannon diversity index for each sample was calculated in Excel via the formula −∑(pi⋅ln(pi)). To avoid errors from ln(0), zero values were replaced with 0.001 as a correction. To analyze differences in the Shannon index, we used R software (version 4.5.0 (2025-04-11); The R Foundation for Statistical Computing, Vienna, Austria) and RStudio (version 2025.05.0+496). The data are presented as the means with 95% CIs.

The Shannon index values (mean [95% CI]) were as follows: males 20–29 years (*n* = 268), 1.86 [1.84–1.89]; males 30–39 years (*n* = 253), 1.91 [1.88–1.94]; males 40–49 years (*n* = 221), 1.97 [1.94–2.00]; males 50–59 years (*n* = 179), 2.00 [1.98–2.03]; females 20–29 years (*n* = 878), 1.94 [1.93–1.96]; females 30–39 years (*n* = 301), 2.00 [1.98–2.02]; females 40–49 years (*n* = 336): 2.02 [2.00–2.03]; females 50–59 years (*n* = 307), 2.03 [2.02–2.05] (Figure 1).

Figure 1 shows that the difference in the Shannon index between males and females decreased over time, and the potential interaction between age and sex was examined.

A Type III ANOVA of the Shannon index revealed significant main effects of age (*p* < 0.001) and sex (*p* < 0.001). However, the interaction effect between age and sex was not statistically significant but tended toward significance (*p* = 0.081). Thus, since the possibility of an interaction between age group and sex remains, we considered it appropriate to evaluate each age × sex combination separately rather than treating age and sex each as a single main effector.

### 3.4. Investigation of Dietary Patterns Focusing on Beta Diversity

The Shannon index, an alpha diversity index, reflects the uniformity and number of types of food consumed and does not directly reflect the trends or patterns of intake for specific food groups. Therefore, in this study, after determining the differences in alpha diversity among groups, NMDS and RDA were conducted on the differences in the patterns themselves.

#### 3.4.1. Visualization of Dietary Patterns with NMDS

**Figure 2:**
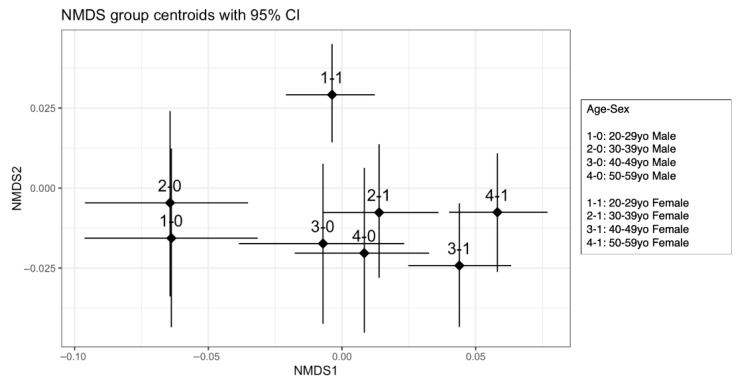
Nonmetric multidimensional scaling (NMDS) of dietary patterns in young and middle-aged Japanese participants. NMDS was performed via standardized food group intake data via R software (version 4.5.0 (2025-04-11); The R Foundation for Statistical Computing, Vienna, Austria) and RStudio (version 2025.05.0+496). Dietary intake data for 10 food groups (meat, fish, egg, soy, milk, seaweed, vegetable, fruit, potato, and oil) were used, and groups were defined by combinations of age and sex (1-0: 20–29 y/o male, 2-0: 30–39 y/o male, 3-0: 40–49 y/o male, 4-0: 50–59 y/o male, 1-1: 20–29 y/o female, 2-1: 30–39 y/o female, 3-1: 40–49 y/o female, 4-1: 50–5 9 y/o female). Bray–Curtis dissimilarity matrices were calculated via the vegdist function, and NMDS was performed in two dimensions (metaMDS function, k = 2, trymax = 100). For each age–sex group, the centroid (mean NMDS1 and NMDS2 values) and 95% confidence intervals were estimated via nonparametric bootstrapping with 2000 replicates (boot package). The data are represented as centroids with 95% CIs.

NMDS is a method of compressing high-dimensional data to a lower dimension while preserving the relative distances between individuals. In other words, it preserves only the greater or lesser distances and ignores the absolute distances between individuals. This makes it possible to investigate patterns in the data in nonmetric spaces. Figure 2 shows that the centroids with 95% CIs in the age–sex groups are displayed in the NMDS. From the visualized figures, women in their 20s and women in their 30s, 40s, and 50s; men in their 20s or 30s; men in their 40s and 50s; men and women in their 20s; and men and women in their 30s were visibly distant from each other. Thus, although a causal relationship is not known, dietary patterns may exist when individuals are grouped by age and sex.

#### 3.4.2. The RDA Results Suggested That Dietary Patterns Varied by Age and Sex

Since the Shannon index and NMDS indicated that dietary patterns may differ by age and sex, we wanted to further clarify the relationships between age, sex, and dietary patterns. RDA is an analysis that combines multivariate analysis and PCA using a response variable (dietary pattern consisting of the frequency of 10 items) and explanatory variables (categorical variables of age and sex).

**Figure 3:**
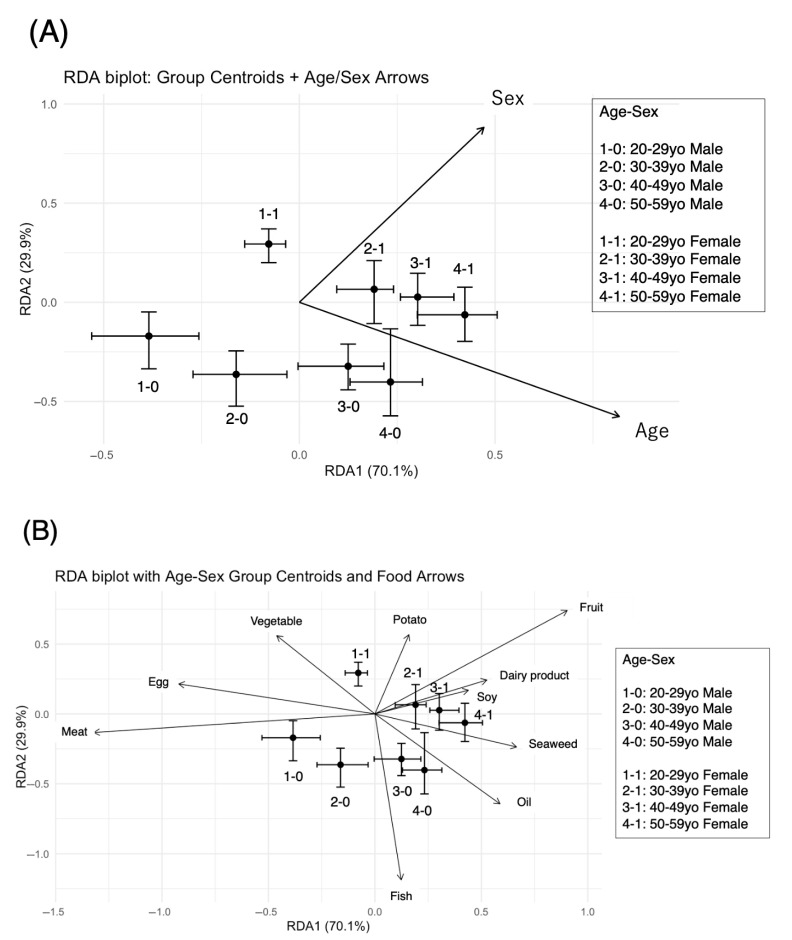

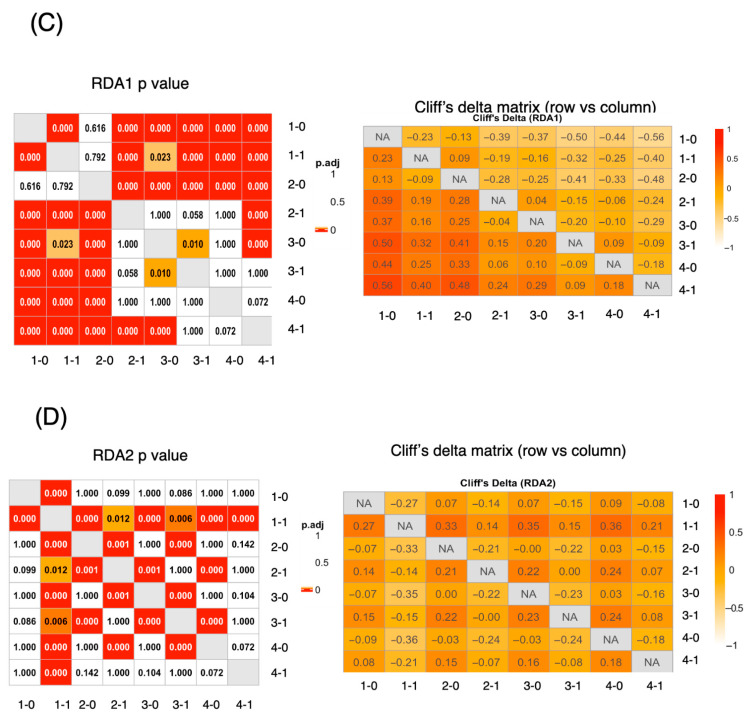
RDA of dietary patterns in young and middle Japanese participants. (**A**) RDA biplot: group centroids with 95% CI + Age/Sex arrows. (**B**) RDA biplot: group centroids with 95% CIs + food arrows. All multivariate analyses were conducted via R software. Dietary intake data for 10 food groups (meat, fish, egg, soy, milk, seaweed, vegetable, fruit, potato, and oil) were used, and groups were defined by combinations of age and sex (1-0: 20–29 y/o male, 2-0: 30–39 y/o male, 3-0: 40–49 y/o male, 4-0: 50–59 y/o male, 1-1: 20–29 y/o female, 2-1: 30–39 y/o female, 3-1: 40–49 y/o female, 4-1: 50–59 y/o female). The dietary intake data for 10 food groups were standardized via Z score transformation and used as dependent variables. Age and sex were included as independent variables in the RDA model via the rda() function. Sample scores (site scores) were extracted, and group labels (according to age and sex) were assigned. Groupwise medians (centroids) and 95% confidence intervals in the RDA space were estimated via nonparametric bootstrapping with 2000 replicates (boot package). Visualization of the redundancy analysis (RDA) was performed via the ‘vegan’ package in R. Arrows indicate food contributions, and group centroids were overlaid with 95% confidence intervals. In some plots, the biplot scores of explanatory variables (from ‘display = “bp”’) were also visualized as vectors to illustrate the influence of age and sex. Data are represented as centroids of the median with 95% CI. Summary statistics for RDA1 and RDA2 scores are also provided in Table 4. (**C**) RDA1 *p* value and Cliff’s delta. (**D**) RDA2 *p* value and Cliff’s delta. To assess between-group differences along the first and second RDA axes (RDA1 and RDA2), Kruskal-Wallis tests were first conducted. When significant differences were found, post hoc comparisons were performed via Dunn’s test with Bonferroni correction. In addition to *p* values, effect sizes were calculated via Cliff’s delta for all pairwise group comparisons. This nonparametric metric quantifies the magnitude of the difference and provides 95% confidence intervals. The magnitude of Cliff’s delta (|δ|) can be interpreted as follows: values between 0.00 and 0.147 indicate a negligible effect, values from 0.147 to 0.33 represent a small effect, values from 0.33 to 0.474 indicate a medium effect, and values equal to or greater than 0.474 are considered large effects.

To clarify the contribution of food groups to age- and sex-related differences, we conducted RDA adjusted for age and sex. The model explained 2.01% of the variance (adjusted R^2^ = 1.94%), with age and sex accounting for 56.7% and 43.3% of the explained variance, respectively (Table 3). RDA1 and RDA2 were modestly correlated with age (r = 0.26, −0.14) and sex (r = 0.15, 0.21) (Table 3 and Figure 3A). Permutation tests (*n* = 999) confirmed significant effects for both age (variance = 0.114, F = 31.95, *p* = 0.001) and sex (variance = 0.087, F = 24.36, *p* = 0.001). The first and second RDA axes explained 1.41% and 0.60% of the total variance in food group intake, respectively (Figure 3A). Within the constrained space, RDA1 and RDA2 explained 70.1% and 29.9% of the constrained variance, respectively (Table 3). The VIFs were 1.04 for sex (female), 1.22 for the 30–39-year-old age group, 1.20 for the 40–49-year-old age group, and 1.18 for the 50–59-year-old age group, thus avoiding the multicollinearity problem.

The distribution of samples in the RDA space (RDA1 and RDA2) was visualized, with each point representing an individual, colored by group (Figure 3A,B, Table 4). Group centroids and their 95% confidence intervals are also indicated to illustrate group differences and overlaps in dietary patterns (Figure 3A,B, Table 4). Thus, the contribution of the RDA1 axis was greater for males and older adults, whereas the contribution of the RDA2 axis was greater for females and younger adults (Figure 3A). Consistent with the results of the Shannon index, the distance between men and women in the same age group decreased with age (Figure 3A). The biplot scores indicate that increasing age is strongly associated with the RDA1 axis (r = 0.817), shifting dietary patterns to the right in the RDA space. Sex (coded as 1 = female) is positively associated with both RDA1 and RDA2, suggesting that females are located in the upper-right quadrant of the plot (Table 5).

Next, we examined the contribution of diet to RDA1 and RDA2; on the RDA1 axis (70.1% contribution), “meat” and “eggs” had very strong negative contributions (−0.91 and −0.63), whereas “fruits,” “seaweed”, and “dairy products” had moderate to strong positive contributions (0.62, 0.46, 0.40) (Figure 3B and Table 6). On the other hand, on the RDA2 axis (29.9% contribution), “fish” had an overwhelmingly strong negative contribution (−0.66), whereas “fruits,” “green and yellow vegetables”, and “potatoes” had strong positive contributions (0.41, 0.31, and 0.32, respectively). Thus, RDA1 was interpreted as a strong reflection of the “animal food-centered (meat and egg) vs. fruit and dairy-centered eating pattern” conflict, whereas RDA2 was interpreted as the axis of the “fish-centered eating pattern (fruit/seaweed/dairy product)” and “plant food-centered eating pattern (fruit/potato/vegetable)” (Figure 3B and Table 6). RDA1, which reflects a meat/egg-centered pattern vs. a fruit/seaweed/dairy product-centered pattern, was lowest in the younger male group and highest in the older female group. On the other hand, RDA2, which reflected a Japanese dietary pattern of (negative) fish- and fat-driven foods and (positive) a traditional pattern of mainly plant-based foods (fruit/potato/vegetable), was highest in the older male group and lowest in the younger female group (Figure 3B and Table 6). Thus, among the food groups, fruit (1.035) and meat (0.984) showed the strongest contributions to the separation of dietary patterns along the RDA axes. Fruit loaded positively on both RDA1 and RDA2, while meat was strongly negatively associated with RDA1. These directions correspond to opposing dietary trends across age and sex groups (Table 6).

Next, the Dunn test and Cliff delta test were conducted for comparisons between groups (Figure 3C,D). The RDA1 *p* value revealed that the patterns for males in their 20s were not significantly different from those for males in their 30s (*p* = 0.616) but were significantly different from those for males in their 40s and 50s (*p* < 0.0001), and the patterns for females in their 20s were significantly different from those for females in their 30s, 40s, and 50s (*p* < 0.0001, *p* < 0.0001, *p* < 0.0001, respectively) (Figure 3C). In the comparison between men and women, significant differences were observed between men and women in their 20s, 30s, and 40s (*p* < 0.0001, *p* < 0.0001, *p* = 0.010, respectively) but not between men and women in their 50s (*p* = 0.072) (Figure 3C). The Cliff delta absolute values for RDA1 were 0.13, 0.37, and 0.44 for men in their 30s, 40s, and 50s, respectively, compared with men in their 20s, indicating negligible, moderate, and moderate effects, respectively. Compared with women in their 20s, the Cliff delta absolute values for RDA1 in their 30s, 40s, and 50s were 0.19, 0.32, and 0.40, respectively, which were small, moderate, and moderately large, whereas for men and women in their 20s, 30s, 40s, and 50s, the effects were 0.23, 0.28, 0.20, and 0.18, respectively (Figure 3C). The *p* values for RDA2 were not significantly different for males in their 20s from those for males in their 30s, 40s, and 50s (*p* = 1.0, *p* = 1.0, and *p* = 1.0, respectively) (Figure 3D). On the other hand, there was a significant difference between women in their 20s and those in their 30s, 40s, and 50s (*p* = 0.012, *p* = 0.006, and *p* < 0.0001, respectively) (Figure 3D). The Cliff delta of RDA2 significantly differed between males and females in their 20s, 30s, and 40s (*p* < 0.0001, *p* = 0.001, and *p* < 0.0001, respectively) but not between males and females in their 50s (*p* = 0.072) (Figure 3D). Compared with those of males in their 20s, the cliff deltas of RDA2 were 0.07, 0.07, and 0.09 for males in their 30s, 40s, and 50s, respectively, and 0.14, 0.15, and 0.21 for females in their 30s, 40s, and 50s, respectively, compared with those of females in their 20s. For males and females in their 20s, 30s, 40s, and 50s, the effects were 0.27, 0.21, 0.23, and 0.18, respectively (Figure 3D). Thus, with respect to dietary patterns, the results showed that the differences between men and women were greater at younger ages, and for the same sex, they were greater at older ages. In addition, meat, fish, fruits, eggs, seaweed, dairy products, vegetables, and potatoes are suggested to influence differences in dietary patterns.

## 4. Discussion

Although it has been empirically demonstrated that dietary diversity varies by age and sex, few studies have revealed significant differences in terms of patterns. Therefore, the aim of this study was to clarify the differences in dietary patterns among young and middle-aged Japanese individuals by age group and sex via statistical methods such as alpha diversity and beta diversity analyses. The alpha diversity (Shannon index) was greater in females than in males and increased with age.

The difference in the Shannon index between men and women decreased with age. Consistent with these findings, the interaction between age and sex tended to affect the Shannon index. While this *p*-value does not meet the conventional threshold for statistical significance (*p* < 0.05), it may still suggest a potentially meaningful trend, especially in the context of biological plausibility and prior literature supporting sex- and age-related differences. As people get older, the proportion of those living with their families increases, so it is empirically understood that the differences in diet between men and women tend to decrease due to eating meals together at home. In fact, although we have not conducted statistical analysis, data we previously reported also showed that the differences in consumption frequency between men and women for eggs, seaweed, fruit, potatoes, and oils became smaller compared to those in their 20s. A larger sample size may be needed to confirm the interaction effect with greater statistical power.

Furthermore, differences in dietary patterns by sex and age were also observed in the NMDS. The RDA method was subsequently used to determine what food intake frequencies were involved in the dietary patterns of the age and sex groups. The contribution of the age–sex interaction was significant, although only approximately 2%, with 56.7% of the effect contributed by age and 43.3% contributed by sex. The burden of food intake frequency on RDA1 and RDA2 was then examined. On the RDA1 axis (1.4%, variance explained within constrained space 70.1%), “meat” and “eggs” had very strong negative contributions (−0.91 and −0.63), whereas “fruit”, “seaweed”, and “dairy products” had moderate to strong positive contributions (0.62, 0.46, 0.40). On the other hand, on the RDA2 axis (0.6%, variance explained within constrained space 29.9%), “fish” had an overwhelmingly strong negative contribution (−0.66), whereas “fruits”, “green and yellow vegetables”, and “potatoes” had strong positive contributions (0.41, 0.31, 0.32). These results suggest that the dietary patterns of the groups differed by age and sex are defined by the frequency of intake of meat, fish, eggs, fruits, seaweed, dairy products, vegetables, and potatoes. Analysis of α-diversity and β-diversity revealed differences in dietary patterns by age and sex, with differences between sexes decreasing with age. In addition, meat, fish, eggs, fruits, seaweed, dairy products, vegetables, and potatoes are suggested to influence the differences in dietary patterns.

We calculated the Shannon index in this study to examine the dietary diversity of individuals. The Shannon index is used as a parameter of individual diversity [20]. Cassol et al. reported that alpha diversity can be categorized into the following groups: richness, measured by the Chao1, ACE, Fisher, Margalef, Menhinick, observed species, and Robbins indices; dominance (also known as evenness), measured by the Berger–Parker, dominance, Simpson, ENSPIE, Gini, McIntosh, and Strong indices; phylogenetics, measured by the Faith index; and information, followed by the Shannon, Brillouin, Heip and Pielou indices. In microbiome studies, the Shannon index reflects the number of different microbes present in a sample and the evenness of their distribution within the sample, encompassing and integrating richness and dominance information. The greater the number of microbes in a sample is, the greater the value of the Shannon index is, and the lower the inequality of relative abundances is, the greater the Shannon index is [20]. Thus, the Shannon index can be used to evaluate both the number and evenness of individual food intake frequencies, and it is reasonable to use the Shannon index for individual food intake diversity. Given that there are trace elements and vitamins tied to each food (e.g., pork and vitamin B1, fish meat and vitamin B12), it may be useful to compare the Shannon index to deficiencies in dietary balance (trace elements and vitamins) as an indicator of individual diversity.

In our study, the Shannon index was greater for women than for men, i.e., the dietary patterns of females were more diverse than those of males. Furthermore, with increasing age, the Shannon index greatly increased. The dietary diversity was lowest among males in their 20s, but the difference between the sexes was greater among those in their 50s. Consistent with our results, Takabayashi et al. reported that, according to the DDS, which scores the frequency of intake of 11 foods (including cereals in addition to the 10 foods in our study), dietary diversity was actually greater for women than for men in the 20–50-year age group and for older individuals than for younger individuals [21]. The diversity was greater for women than for men and for those of older age than for those of younger age in the 20–50-year age group [21]. Previous reports have also shown that with increasing age, the frequency of the intake of many foods, including soy, seaweed, dairy products, vegetables, fruits, potatoes, fish, and oils, increases with age [4]. Therefore, we considered it reasonable that dietary diversity would be greater for women than for men and in older age groups than in younger age groups.

This result was also observed to be true by RDA, adjusted for age and sex, with the contribution of RDA1, which reflects the (negative) meat and egg-centered vs. (positive) fruit and dairy product-centered patterns, being greater in the younger male group and possibly lowest in the older female group. In contrast, the contribution of RDA2, reflecting (negative) fish- and fat-driven Japanese food vs. (positive) plant-based (vegetable, fruit, and potato) traditional food patterns, was lowest in the older male group and highest in the younger female group. This may reflect the fact that, as people in their 20s live alone and eat out more often, they have more opportunities to eat meat at home and consume more fish and dairy products. Consistent with these findings, the Japanese Ministry of Health and Welfare’s National Health Survey also revealed that fruit and vegetable intake increases with age and is higher among women than among men [22]. Therefore, the differences in the patterns of age and sex groups according to the RDA and the contribution of foods were considered reasonable.

Although the total variance explained by the RDA model was relatively low (R^2^ = 2.01%), this was not uncommon in the ecological and nutritional data due to high individual variability. For example, it is common for R2 values to be low (below 10%) in community ecology research [23]. R-squared measures how much of the variation in the dependent variable is explained by the independent variable(s) in a regression model. In ecological studies, many factors can influence community composition, making it difficult for a single model to perfectly explain all the variance. Similarly, dietary patterns are influenced by many factors in addition to age and gender, including region, income, income, urbanization, trade liberalization, family structure, occupation, and education [24,25,26,27]. In this study, both explanatory variables (age and sex) significantly contributed to the constrained ordination space, as confirmed by permutation tests (*p* = 0.001). The constrained axes (RDA1 and RDA2) together captured meaningful patterns in the data structure, with RDA1 accounting for 70.1% of the constrained variance. Therefore, these results suggest that, despite the modest total variance explained, age and sex play substantial roles in shaping dietary patterns.

One limitation of this study is that it was a cross-sectional observational study. Second, the target population included many healthy individuals with high medical literacy. In addition, possibly due to the professional composition, there are many women and a relatively younger demographic. Since this is a single-institution study, caution should be exercised when generalizing the results to the Japanese population as a whole. On the other hand, based on these results, we aim to improve the nutritional status of people working in our own workplace, and this aligns with our objective of utilizing the findings for health management. In addition, this population was limited to those without underlying medical conditions, and further study is needed to determine whether the results apply to those with underlying medical conditions such as diabetes. There were twice as many women as men in the group, and the cohort was also skewed with respect to age. Importantly, the age–sex interaction explained only 2% of the difference among the groups. Since this RDA was limited to the explanatory contributions of age and sex, variations due to other factors remained as residual variance. For example, even among such a medically literate population, dietary diversity was low at younger ages, suggesting that this problem cannot be solved by simply raising awareness of dietary content. As the age group increases, family size tends to be larger, and in the context of universities, higher age groups generally have higher incomes and greater economic prosperity. Furthermore, among people working at universities, the older they are, the higher their final academic background tends to be. Therefore, economic factors, family structure, educational background, and other variables that fluctuate with age can be considered confounding factors that may have influenced the results of this study. Therefore, in future studies, multivariate analyses that include other explanatory variables will be important. In an analysis of a broader population, the inclusion of factors such as income, smoking history, alcohol consumption, and family structure (living alone vs. family) may also be necessary. However, several statistical considerations exist regarding the analysis. The Kruskal–Wallis and Dunn tests (corrected for this value) were used as nonparametric methods to detect significant differences between groups, but as the number of groups increased, the power of the tests decreased owing to multiple comparison correction. Therefore, we referred to Cliff’s delta, as well as the presence or absence of significant differences.

## 5. Conclusions

In conclusion, in this study, age- and sex-related differences in dietary patterns, which until now had been broadly recognized as differences in food intake, were clearly captured statistically. These results can be used to provide dietary guidance for nutrient-deficient individuals in each age group. Since this technique has been used in studies of intestinal bacteria for some time, a comparison of intestinal bacterial diversity and dietary diversity will be made in the future. In addition, this technique could be used to predict diet-related diseases. Furthermore, when age- and sex-matched, we compared dietary patterns between those who developed diseases such as diabetes and cancer and those who did not. In addition to age and sex, socioeconomic factors that influence dietary patterns must be considered. The influence of socioeconomic factors on dietary patterns may be particularly important for those aged 60 years and older, who were not included in the current study. In conclusion, the analysis of α diversity and β diversity seemed meaningful in clarifying the role of factors (age and sex) related to dietary patterns in the Japanese population. Nutritional guidance must account for variations in dietary patterns influenced by age and sex.

## Figures and Tables

**Table 1:** Characteristics of the participants in this study.

	Total (*n* = 2743)	Male (*n* = 921)	Female (*n* = 1822)
Age (years old)	36.05 (11.39)	38.24 (10.73)	34.94 (11.56)
BMI (kg/m^2^)	22.05 (3.45)	23.30 (3.45)	21.42 (3.27)
HbA1c (%)	5.45 (0.35)	5.48 (0.40)	5.44 (0.33)
eGFR (mL/min/1.73 m^2^)	89.78 (17.86)	85.36 (16.46)	92.02 (18.13)
Total Cholesterol (mg/dL)	190.32 (32.42)	192.60 (33.08)	189.17 (32.02)
Triglyceride (mg/dL)	105.31 (86.31)	135.53 (118.06)	90.03 (58.99)
HDL-C (mg/dL)	62.54 (14.45)	54.87 (13.19)	66.42 (13.49)

Data is represented as mean (SD). Abbreviations: BMI—body mass index; HbA1c—glycated hemoglobin A1c; eGFR—estimated glomerular filtration rate; HDL-C—high-density lipoprotein-C.

**Table 2:** Sex difference in individual food intake frequencies.

	Total (*n* = 2743)	Male (*n* = 921)	Female (*n* = 1822)	*p*	Cliff‘s Delta (Male vs. Female)
Meat	8.0 (6.0–11.0)	9.0 (6.0–12.0)	8.0 (5.0–11.0)	<0.001	0.099
Fish	4.0 (2.0–6.0)	4.0 (2.0–6.0)	4.0 (2.0–6.0)	<0.001	0.11
Egg	4.0 (3.0–6.0)	4.0 (3.0–7.0)	4.0 (3.0–6.0)	0.83	0.005
Soy	5.0 (3.0–8.0)	5.0 (2.0–8.0)	5.0 (3.0–8.0)	0.0036	−0.068
Dairy product	5.0 (2.0–10.0)	4.0 (1.0–9.0)	5.0 (2.0–10.0)	<0.001	−0.11
Seaweed	2.0 (0.0–3.0)	2.0 (0.0–3.0)	2.0 (0.0–3.0)	0.086	−0.039
Vegetable	8.0 (6.0–12.0)	8.0 (5.0–11.0)	8.0 (6.0–12.0)	0.0059	−0.064
Fruit	2.0 (1.0–5.0)	2.0 (0.0–4.0)	3.0 (1.0–5.0)	<0.001	−0.19
Potato	2.0 (1.0–3.0)	2.0 (1.0–3.0)	2.0 (1.0–3.0)	<0.001	−0.10
Oil	11.0 (7.0–15.0)	11.0 (7.0–15.0)	11.0 (7.0–15.0)	0.98	−0.0007

Data (times per weeks) is represented as median (IQR). The magnitude of Cliff’s delta (|δ|) can be interpreted as follows: values between 0.00 and 0.147 indicate a negligible effect, values from 0.147 to 0.33 represent a small effect, values from 0.33 to 0.474 indicate a medium effect, and values equal to or greater than 0.474 are considered large effects. Oil refers to edible fats and oils, including butter, margarine, and cooking oils, among others.

**Table 3:** Summary of the redundancy analysis (RDA) model.

	Value
Total variance explained (R^2^)	2.01%
Adjusted R^2^	1.94%
Variance explained by RDA axes	
-RDA1	1.41%
-RDA2	0.60%
Variance explained within constrained space	
-RDA1	70.1%
-RDA2	29.9%
Contribution of each predictor	
-Age	56.7%
-Sex	43.3%
Correlation between predictors and RDA axes	
-RDA1—Age	r = 0.26
-RDA2—Age	r = −0.14
-RDA1—Sex	r = 0.15
-RDA2—Sex	r = 0.21
Permutation test results (*n* = 999)	
-Age: F = 31.95, Variance = 0.114	*p* = 0.001
-Sex: F = 24.36, Variance = 0.087	*p* = 0.001

**Table 4:** Age–sex group centroids with 95% confidence intervals.

	RDA1	RDA2
20–29 y/o male	−0.38 (−0.53, −0.26)	−0.17 (−0.34, −0.049)
30–39 y/o male	−0.16 (−0.27, −0.032)	−0.36 (−0.52, −0.25)
40–49 y/o male	0.13 (−0.003, 0.22)	−0.32 (−0.44, −0.21)
50–59 y/o male	0.23 (0.13, 0.32)	−0.40 (−0.57, −0.13)
20–29 y/o female	−0.078 (−0.14, −0.035)	0.29 (0.20, 0.37)
30–39 y/o female	0.19 (0.095, 0.24)	0.065 (−0.11, 0.21)
40–49yo female	0.30 (0.26, 0.40)	0.026 (−0.12, 0.15)
50–59 y/o female	0.42 (0.30, 0.51)	−0.063 (−0.20, 0.076)

Data represented as median with 95% CI.

**Table 5:** Standardized biplot scores of explanatory variables (scaling = 2).

Predictor	RDA1	RDA2	Interpretation
Age	0.817	−0.576	Age increases along the RDA1 (right) and slightly down RDA2
Sex (1 = female)	0.471	0.882	Female-associated direction is upper-right in RDA space

**Table 6:** RDA biplot scores and vector lengths of food groups.

Food Group	RDA1	RDA2	Vector Length (√(RDA1^2^ + RDA2^2^))
Fruit	0.62	0.41	1.04
Meat	−0.91	−0.074	0.98
Oil	0.41	−0.36	0.76
Egg	−0.64	0.12	0.75
Fish	0.085	−0.66	0.75
Vegetable	−0.32	0.31	0.63
Seaweed	0.46	−0.13	0.59
Dairy product	0.36	0.14	0.5
Potato	0.11	0.32	0.43
Soy	0.3	0.095	0.39

Oil: oils and fats. Blue and red letter imply positive and negative stronger contributors, respectively.

## Data Availability

Some or all datasets generated and/or analyzed during the current study are not publicly available due to their containing information that could compromise the privacy of research participants but are available from the corresponding author upon reasonable request.
